# Novel Insight into *Culicoides* (Diptera: Ceratopogonidae) Host Preferences and the First Evidence of Avian Haemosporidian Parasites in Biting Midges in Slovakia

**DOI:** 10.3390/pathogens14060515

**Published:** 2025-05-22

**Authors:** Nikola Janošková, Andrea Schreiberová, Ľuboš Korytár, Lenka Minichová, Alica Kočišová

**Affiliations:** 1 Department of Epizootiology, Parasitology and Protection of One Health, University of Veterinary Medicine and Pharmacy in Košice, Komenského 73, 041 81, Košice, Slovakia; 2 Institute of Virology, Biomedical Research Centre, Slovak Academy of Sciences, Dúbravská cesta 9, 845 05 Bratislava, Slovakia; 3 Institute of Microbiology, Czech Academy of Sciences, Víděnská 1083, 142 00 Prague, Czech Republic

**Keywords:** biting midges, molecular detection, host blood, potential vectors

## Abstract

*Culicoides* biting midges (Diptera: Ceratopogonidae) are important vectors of avian haemosporidian parasites. Understanding their host preferences is crucial for elucidating transmission routes of vector-borne pathogens. In Slovakia, such knowledge is limited, particularly in forested wetlands. This study aimed to identify *Culicoides* species, their host preferences, and haemosporidian parasites in a wetland ecosystem at the Bird Ringing Station in Drienovec. Midges were collected in 2022 using UV light traps at two sites. In total, 2344 *Culicoides* individuals of 19 species were collected. Host blood was identified and DNA subsequently extracted from 36 engorged females, revealing feeding on three mammal and five bird species. The most frequently identified host was roe deer (*Capreolus capreolus*), predominantly fed upon by *Culicoides obsoletus* (Meigen 1818). Notably, avian haemosporidian DNA was detected for the first time in Slovakia in three *Culicoides* females. In two *Culicoides alazanicus* Dzhafarov 1961 individuals, DNA of *Haemoproteus asymmetricus* (TUPHI01) and *Plasmodium matutinum* (LINN1) was confirmed, both associated with avian blood from *Turdus* sp. One *Culicoides festivipennis* Kieffer 1914 female carried *Haemoproteus tartakovskyi* (HAWF1) and fed on *Coccothraustes coccothraustes*. These findings highlight the potential role of local *Culicoides* species in transmitting avian pathogens and underscore the importance of monitoring their ecology.

## 1. Introduction

*Culicoides* biting midges (Diptera: Ceratopogonidae) are small haematophagous flies, which, with the exception of Antarctica and New Zealand, have a worldwide distribution [1]. The Ceratopogonidae family comprises more than 6270 species; of which, as much as 1399 species belong to the *Culicoides* genus [2]. In Slovakia, 65 species have been described so far [3,4,5]. Certain species are regarded as vectors of multiple pathogens, including viruses, bacteria, and parasites [2]. Ornithophilic species of biting midges are implicated in the transmission avian blood parasites (*Haemoproteus*, *Leucocytozoon*, *Plasmodium,* and *Trypanosoma*), which cause diseases in domestic and wild birds [6,7,8,9]. *Culicoides* play an essential role in the parasite–host relationships in various ecosystems and may have a significant economic impact [1,9,10,11]. Identification of host blood from engorged females of biting midges is crucial for the understanding of the transmission of blood parasites and their role as vectors. In multiple scientific studies, avian hosts were detected [12,13] in engorged females of *Culicoides*, such as *C. circumscriptus* Kieffer 1918, *C. duddingstoni* Kettle and Lawson 1955, *C. salinarius* Kieffer 1914, *C. kibunensis* Tokunaga 1937 (syn. *C. cubitalis* Edwards)*,* and *C. pictipennis* (Staeger 1839), by the molecular identification of host blood meal [14,15,16]. Other species, for example, *C. impunctatus* Goetghebuer 1920, *C. chiopterus* (Meigen 1830)*,* and *C. deltus* Edwards 1939, are regarded as mammophilic as they exhibit a host preference for bigger mammals [16,17,18]. Certain species are non-host-specific, for example, *C. festivipennis*, *C. pulicaris* Linnaeus 1758, and *C. obsoletus* [12]. The majority of studies involving host preferences of biting midges dealt with livestock, which were kept on farms (sheep and cattle) or lived near them, while less attention was paid to ornithophilic species [1,15]. Such an approach may create a gap in the knowledge related to the risk of transmission of blood parasites in wild nature [19], since there is a lack of information about the *Culicoides* species that live in forest biotopes, wetlands, and other anthropogenically unaffected regions where the diversity of wild vertebrates is higher [20]. Avian haemosporidioses is caused by single-cell parasites, which are members of the largest group of the Haemosporida order as to the number of species already described [21]. To date, more than 200 species of haemosporidian have been described and assigned to three key genera: *Plasmodium*, *Haemoproteus*, and *Leucocytozoon* [6]. In Europe, 15 *Culicoides* species have been identified as potential vectors of avian haemosporidia *C. alazanicus* Dzhafarov (syn. *C. musilator*), *C. circumscriptus*, *C. festivipennis*, *C. impunctatus*, *C. kibunensis*, *C. obsoletus*, *C. pictipennis*, *C. punctatus* (Meigen 1804), *C. segnis* Campbell and Pelham-Clinton 1960, *C. scoticus* Downes and Kettle 1952, *C. paolae* Boorman 1996, *C. reconditus* Campbell and Pelham-Clinton 1960, *C. pallidicornis* Kieffer 1919, *C. simulator* Edwards 1939, and *C. pulicaris* [22,23,24,25,26,27,28,29,30,31,32]. The purpose of the present study was to identify the species composition of biting midges, their host preferences, as well as the occurrence of avian haemosporidian parasites at the Bird Ringing Station in Drienovec, south-eastern Slovakia and hotspot of avian biodiversity.

## 2. Materials and Methods

### 2.1. Study Area

**Figure 1:**
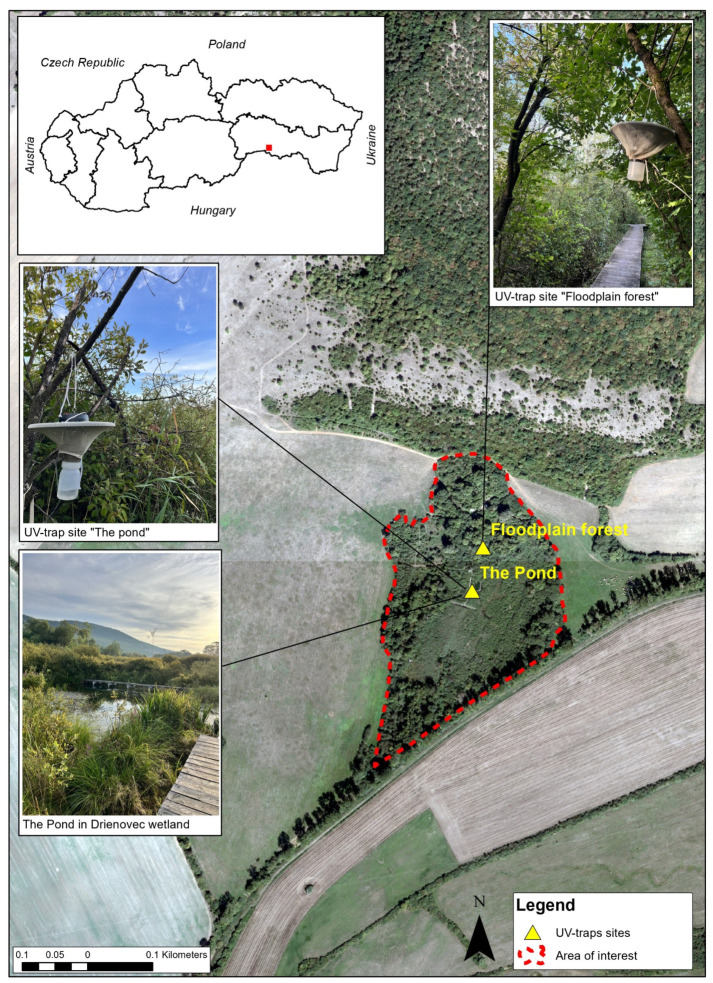
Location of Drienovec Wetland with UV traps for collecting *Culicoides* biting midges. Legend: Red lines depict the border of the study area. Yellow triangles depict the placement of UV traps. The insert in the upper left corner shows the location of Slovakia in relation to its neighbouring countries.

The entomological research was conducted at the Bird Ringing Station, located in the Drienovec Wetland (48°10′ N, 17°03′ E) in the south-eastern part of Slovakia, as part of a pilot avian haemosporidian parasites programme. In terms of orography, the region lies directly on the border between the Košická Kotlina basin and the Slovak Karst National Park, at an altitude of 181 metres above sea level, with a total surface area of 7.7 ha, far from the city and with minimal human interference [33]. On this relatively small area, there is a wide range of ruderal biotopes with bushes, sedges, and canes with free water surface. In the north, the location is surrounded by a steep slope of the Jasovská Planina plateau of a typical karstic nature (at an altitude of 500–700 m above sea level) with mostly oaks and turkey oak forest. The average annual temperature in the study area is 8.5 °C; however, during the peak activity period of *Culicoides* midges, the average air temperature in July reaches 19–20 °C, with a mean monthly precipitation of 60–80 mm [34]. Research on bird migration has been ongoing since 1998. Since 2006, systematic monitoring of the breeding community has been carried out from the beginning of May to the end of July, with a focus on avian migration along the south-eastern African–Eurasian Flyway. Dominant bird species in the region include passerines such as *Phylloscopus collybita* (common chiffchaff), *Sylvia atricapilla* (blackcap), *Erithacus rubecula* (European robin), and thrushes like *Turdus merula* (blackbird) and *Turdus philomelos* (song thrush). Additionally, woodpeckers such as *Dendrocopos major* (great spotted woodpecker) and *Dendrocopos minor* (lesser spotted woodpecker), as well as raptors like *Buteo buteo* (common buzzard) and *Accipiter nisus* (sparrowhawk), are commonly found. The region also hosts larger mammals like wild boar (*Sus scrofa*), roe deer (*Capreolus capreolus*), and foxes (*Vulpes vulpes*), contributing to the area’s ecological dynamics [35]. The collection sites were chosen based on the presence of a wetland and enclosed forest and the absence of wind since those factors determine suitable conditions for the presence and reproduction of various species of *Culicoides* (Figure 1).

### 2.2. Culicoides Collection and Processing

*Culicoides* were collected from 4 June to 30 July, every 10 days during the summer of 2022, using special 12 V UV light traps, CDC Miniature Trap 1212 (John Hock Company, Gainesville, FL, USA), installed at a height of 112 1.5–2 m above ground level. To increase the numbers collected as well as species diversity, collections were made at two locations, the pond and the floodplain forest, in the area (Figure 1). The traps were installed approximately one hour before sunset and checked for the presence of insects the next day at dawn. The traps equipped with a collection container containing 50% ethanol, in which the trapped insects were preserved. The insects were transported to a laboratory, where they were transferred to 70% ethanol until further determination and diagnostics. The specimens were stored at 4 °C.

### 2.3. Laboratory Processing of Biting Midges and Their Morphological Diagnostics

**Figure 2:**
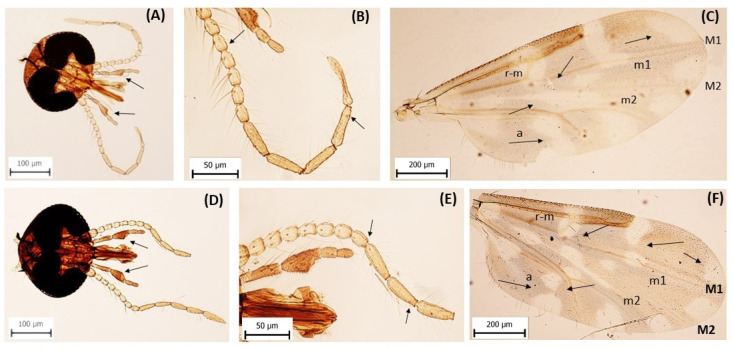
The head (**A**), antenna (**B**), and wing (**C**) of *Culicoides clastrieri* and head (**D**), antenna (**E**), and wing (**F**) of *Culicoides festivipennis*. Both species exhibit similar head structures, including broadly contiguous eyes and a triangular, moderately swollen third palpal segment. In *C. festivipennis*, sensilla coeloconica are present on antennal segments III to XV, whereas in *C. clastrieri*, they occur only on segments III and XI to XV and are absent on segments VII to X. On the wing of *C. festivipennis*, cell m2 has a single pale spot, cell m has two to three separate pale spots, and the anal cell has two distal pale spots. On the wing of *C. clastrieri*, a pale spot over the r-m crossvein is fused with the m2 spot, forming a continuous area; cell m usually has one fused spot. The anal cell and M1 vein show similar pale spots as in *C. festivipennis*.

In the laboratory, biting midges were separated from the other insects and were morphologically identified using a binocular stereo-microscope (Zeiss-Stemi DV-4, Göttingen, Germany). All biting midges were identified to species or group level and sexed. The *Culicoides* species identified wing spots, the number, and the presence of sensilla coeloconica on the antennas based on the specific shape of 3rd segment of palps, using an interactive key [36]. The *C. festivipennis* and *C. clastrieri* Callot, Kremer, and Deduit 1962, were morphologically differentiated primarily based on the wing spot patterns and presence of sensilla coeloconica (their number and occurrence), since they could not be identified by the DNA barcoding method (Figure 2) [37,38,39]. The specimens depicted in the figures are female. Representative samples of midges were temporarily slide mounted to photograph the specimens. Before being transferred to slides, the wings and heads were removed using micro-preparation needles and briefly rinsed in ethanol to remove any remaining debris. The dissected structures were subsequently mounted in Euparal on microscope slides and positioned to optimise visibility of diagnostic features [36]. Coverslips were carefully applied, and the slides were left to dry at room temperature.

Based on the presence of the burgundy pigment in the abdomens of females, their physiological condition was identified (nulliparous, parous, engorged, or gravid) [40]. The remaining parts were transferred to a 1.5 mL microcentrifuge tube for subsequent DNA isolation.

### 2.4. *DNA* Extraction and Identification of the Culicoides Species

Genome DNA was isolated from the thorax and abdomens of all *Culicoides* females that had fresh blood meal in their abdomens. DNA was isolated from all females that were difficult to identify morphologically. DNA extraction was carried out using a DNeasy Blood commercial set and a Tissue Kit (QIAGEN, GmbH, Hilden, Germany) following the manufacturers’ instructions. The resulting DNA was stored at −20 °C until used for PCR analysis.

The PCR molecular identification of biting midges was based on the sequencing of partial sequences of the mitochondrial cytochrome c oxidase subunit *I* (cox 1) gene, which is used as the identifying DNA barcode for the given species. The partial cox 1 sequences for the individual midges were obtained using forward C1-J-1718 (5′–GGA GGA TTT GGA AAT TGA TTA GT–3′) and reverse C1-N-2191 (5′–CAG GTA AAA TTA AAA TAT AAA CTT CTG G–3′) primers, resulting in a primary product of 550 bp. The amplification procedure included initial denaturation at 95 °C for 5 s, 5 cycles (94 °C, 40 s; 45 °C, 40 s; 72 °C, 60 s), followed by 45 cycles (94 °C, 40 s; 50 °C, 40 s; 72 °C, 1 s), and the final elongation at 72 °C for 7 min [41].

### 2.5. Blood Meal Identification

The molecular identification of host blood from the blood-engorged females was performed by sequencing the 350 bp sequence of the mitochondrial cytochrome *b* (*cyt b*) gene. The universal vertebrate forward primer cyt bb1 (5′-CCA TCM AAC ATY TCA DCA TGA AA-3′) and the reverse primer cyt bb2 (5′-GCH CCT CAG AAT GAY ATT TGK CCT CA-3′) were used for the conventional PCR, while the amplification process consisted of the initial denaturation step at 94 °C for 5 min, followed by 35 cycles at 94 °C for 1 min, at 55 °C for 1 min, at 72 °C for 1 min, and the final step of extension at 72 °C for 7 min [42,43].

### 2.6. Molecular Identification of Avian Haemosporidian Parasites in Vectors

All 46 engorged females were examined for the presence of blood parasites using a nested PCR assay targeting the *cyt b* gene mtDNA, as described by Bensch et al. [44] and Hellgren et al. [45]. Briefly, in the first step, the HaemNFI (5‘-CAT ATA TTA AGA GAA ITA TGG AG-3‘) and HaemNR3 (5‘-ATA GAA AGA TAA GAA ATA CCA TTC-3‘) primers were used to amplify the 617 bp fragment. In the second step, the nested PCR with HaemF (5‘-ATG GTG CTT TCG ATA TAT GCA TG-3‘) and HaemR2 (5′-GCA TTA TCT GGA TGT GAT AAT GGT-3‘) primers amplified the 480 bp fragments of Haemoproteus and Plasmodium. All the reactions were carried out using the GoTaq qPCR Master Mix (Promega, Madison, WI, USA) on a Labcycler (SensoQuest, Goettingen, Germany). The following cycling conditions were used: 95 °C for 2 min; 35 cycles: 95 °C for 30 s, 47 °C for 30 s, 72 °C for 45 s followed by final elongation at 72 °C for 10 min.

Another set of primers was used to confirm the results for haemosporidian parasites; it was recovered using the mitochondrial cytochrome b gene sequences from 52 species and 4 genera (*Plasmodium*, *Hepatocystis*, *Haemoproteus*, and *Leucocytozoom*). In the first step, DW2 (5′-TAA TGC CTA GAC GTA TTC CTG ATT ATC CAG-3′) and DW4 (5′-TGT TTG CTT GGG AGC TGT AAT CAT AAT GTG-3′) primers were used, and 2 μL of genomic DNA was subjected to 35 cycles at 94 °C for 20 s, at 60 °C for 20 s, and at 72 °C for 1.5 min. In the second step, a template from the first step was used for a nested reaction with DW1 (5′-TCA ACA ATG ACT TTA TTT GG-3′) and DW6 (5′-GGG AGC TGT AAT CAT AAT GTG-3′) primers in 40 cycles at 94 °C for 20 s, at 50 °C for 20 s, and at 72 °C for 1 min, and then at 72 °C for 7 min [46]. In every PCR run, positive controls and negative controls (nuclease-free water instead of DNA templates) were used. One negative (nuclease-free water) and one positive (a sample with a single infection of *Haemoproteus parabelopolskyi* from *Sylvia atricapilla*) control was included in each run [47].

All amplified products were visualised on 1% agarose gel under the UV light. Positive PCR products were sent to a Microsynth Seqlab commercial laboratory (Vienna, Austria). Some of the products were sent to SEQme (Dobříš, Czech Republic) or to Macrogen (Amsterdam, The Netherlands) for purification and sequencing in both directions with the use of identical primers. The sequencing was carried out by applying the Sanger sequencing method, and the resulting sequences were analysed and modified using MEGA X software, version 10.1.5, set no. 10191107 [48]. Nucleotide sequences were compiled using Gene Tool Lite version 1.0 software (BioTools Inc., Jupiter, FL, USA). The obtained sequences were compared to the sequences deposit in GenBank by applying BLAST (Basic Local Alignment Search Tool) nucleotide algorithm in the National Centre for Biotechnological Information (NCBI). The sequences obtained in the study were deposited in GenBank under unique accession numbers. The obtained sequences from the DNA of haemosporidian were also compared to the sequences available in the MalAvi database [49,50].

The molecular identification of species and genetic lineages of avian haemospori-dian parasites were further confirmed by phylogenetic analysis. We used nucleotide sequences gene *cyt b* obtained in this study and other haemosporidian parasites available in GenBank NCBI. The sequences were aligned, and the phylogenetic tree of the gene was constructed using MEGA X software version 10.1.5, set no. 10191107 [48]. The phylogenetic analysis was inferred by using the Maximum Likelihood statistical method and Tamura–Nei model with a minimum of 1000 bootstrap replications. The tree with the highest log likelihood (−997.38) is shown (Figure 4). The tree was drawn to scale, with branch length measured in the number of substitutions per site. This analysis involved 10 nucleotide sequences. There were a total of 429 positions in the final dataset.

## 3. Results

### 3.1. Morphological Identification of the Culicoides Species

**Figure 3:**
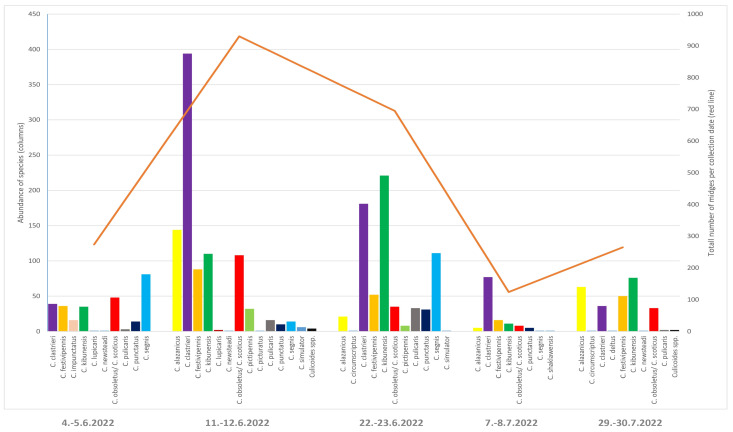
*Culicoides* abundance and species diversity at the Ringing Station Drienovec during June/July 2022. Each one of the five data points reflects the total number of midges collected with two CDC traps. Collections were made overnight at two sites (i.e., a pond and a floodplain forest).

Ten CDC light trap collections were made at two sites at a Bird Ringing station in the Drienovec Wetland in June and July 2022. The total number of collected *Culicoides* species was 2344 individuals, of which only 38 were males. They were morphologically identified as members of 19 species (*C. clastrieri* (*n* = 727/31%); *C. kibunensis* (*n* = 456/19.5%);* C. festivipennis* (*n* = 282/12.4%); *C. alazanicus* (*n* = 239/10.2%); *C. obsoletus/C. scoticus* (*n* = 234/10.0%);* C. segnis* (*n* = 211/9.0%);* C. punctatus* (*n* = 60/2.6%); *C. pulicaris* (*n* = 55/2.4%); *C. pictipennis* (*n* = 40/1.7%); *C. impunctatus* (*n* = 16/0.7%); *C. simulator* (*n* = 6/0.3%); *C. lupicaris* Downes and Kettle 1952 (*n* = 2/0.1%); *C. newsteadi* Austen 1921 (*n* = 2/0.1%);* C. circumscriptum* (*n* = 2/0.1%); *C. picturatus* Kremer and Deduit 1962 (*n* = 1/0.04%); *C. shaklawensis* Wiliams 1955 (*n* = 1/0.04%); *C. deltus* (*n* = 1/0.04%); and *C. pallidicornis* (*n* = 1/0.04%), and eight individuals of the *Culicoides* spp. (*n* = 8/0.3%) were not morphologically identified. Out of the total number of collected biting midges, 46 (1.96%) individuals were engorged females. The highest number of midges and species diversity were found on 11 and 12 June 2022, a total of 930 biting midges (39.67%) were collected, including 29 engorged females with fresh host blood in their abdomens (Figure 3).

### 3.2. Molecular Identification of the Culicoides Species

For the purpose of reliable detection of species composition, randomly selected specimens were identified using the PCR assay. By sequencing a fragment of the *cox 1* gene, 65 individuals out of the total number of biting midges were molecularly confirmed, as shown in Table 1. Based on the BLAST analysis in GenBank, six specimens exhibited similarity below 90%. Five females exhibited 87.47% similarity to the *C. arakawae* Arakawa 1905, while one specimen was 86.96% identical to the *C. oxystoma* Kieffer 1910. The identification of these species requires further studies. Based on the morphological and molecular analyses, a total of 19 species were identified. All of these have been collected previously in Slovakia. The sequences of the mt cox 1 gene of *Culicoides* obtained in this study were deposited in GenBank database under the following accession numbers: PV055075–PV055139 (Table 1).

### 3.3. Blood Meal Analysis of Biting Midges and Detection of Avian Haemosporidian Parasites

The detection and identification of host blood in vectors are essential for understanding the potential of the spread of pathogens. The host DNA was amplified from 36 biting midges out of 46 females containing fresh host blood meal (Table 2).

In ten specimens, DNA probably degraded due to an advanced stage of blood digestion in the midguts of the vectors. In total, the DNA of the host blood of three mammal and five avian species were detected. Females of *C. obsoletus*, *C. clastrieri*, *C. festivipennis*, *C. alazanicus*, and *C. pictipennis* represented the highest number (*n* = 15) of species that fed on roe deer (*Capreolus capreolus*). Human DNA was detected in *C. clastrieri* (*n* = 2), *Culicoides* sp. (*n* = 2), and *C. alazanicus*, and DNA from Eurasian elk (*Alces alces*) was detected in two females of *C. obsoletus* and *C. alazanicus*.

The detected moose DNA was particularly remarkable and surprising, as this species is not endemic to Slovakia, and its occurrence in the country is rather sporadic. Song thrush (*Turdus philomelos*) was the most frequently detected avian host fed on by *C. alazanicus* and *C. clastrieri*, while the second most frequent avian host was the common blackbird (*Turdus merula*), on which *C. clastrieri*, *C. alazanicus,* and *C. pictipennis* fed. In two specimens of *C. alazanicus,* the Turdidae family was detected. In one case, the avian host DNA was detected. Its sequence was compared to GenBank database, and 91.56% similarity was found to the closest relative species of *Turdus viscivorus viscivorus* Mistle thrush. In the second case, the analysed specimen was 91.18% identical to the *Turdus philomelos* species. In one female of *C. festivipennis*, DNA of Eurasian magpie (*Pica pica*) and hawfinch (*Coccothraustes coccothraustes*) were confirmed. A *C. alazanicus* female was found to contain the blood of Eurasian blue tit (*Cyanistes caeruleus ogliastrae*).

Haemosporidian DNA was detected in two *C. alazanicus* and one *C. festivipennis.* In *C. alazanicus,* we detected two species: *Haemoproteus asymmetricus* (TUPHI01) and *Plasmodium matutinum* (LINN1). The DNA of *Haemoproteus tartakovskyi* (HAWF1) was detected in *C. festivipennis* (Table 3). The obtained haemosporidian sequences were compared to the sequences stored in GenBank and MalAvi databases.

### 3.4. Phylogenetic Analysis of Avian Haemosporidian Parasites in Vectors

To examine the phylogenetic relationships between species and genetic lineages of avian haemosporidian parasites, we selected *cyt b* gene sequences from this study—*Haemoproteus tartakovskyi* (HAWF1) host *C. festivipennis*; *Haemoproteus asymmetricus* (TUPHI01) host *C. alazanicus*; and *Plasmodium matutinum* (LINN1) host *C. alazanicus*—as well as reference sequences available in GenBank at NCBI for the same species and lineages. The following sequences of *cyt b* gene were phylogenetically analysed: *H. tartakovskyi* KM361486.2 (host: *Coccothraustes coccothraustes*, from Russia) and DQ368348.1 (host: *Coccothraustes coccothraustes* from Sweden); *H. asymmetricus* OQ311170.1 and OQ311074.1 (host: *Turdus philomelos* from Germany); and *P. matutinum* MT912106.1 and MK652234.1 (host: *Turdus merula* from Austria). A *Hepatocystis* spp. (MW366841.1) sequence from Singapore was used as the outgroup, and bootstrap values were indicated only for nodes with support greater than 50%.

**Figure 4:**
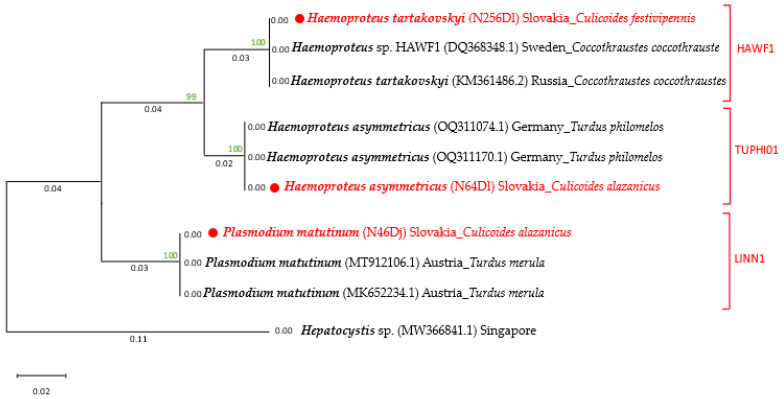
The phylogenetic tree constructed based on *cyt b* gene sequences illustrates the relationships between avian haemosporidian parasites. One sequence of *Hepatocystis* spp. was used as an outgroup. The samples in red represent samples obtained in the present study.

The phylogenetic tree revealed that the sequences of *H. tartakovskyi* and *H. asymmetricus* clustered within the same clade, although they diverged into distinct branches. The sequences of *P. matutinum* were placed in a separate clade. All three species exhibited high homology within their respective groups (Figure 4).

## 4. Discussion

The present study is the first on the occurrence and diversity of biting midges in Slovak Karst National Park. Our study assessed the role of *Culicoides* biting midges as potential vectors of avian haemosporidian parasites in Slovakia. At least nineteen of the sixty-five *Culicoides* species [4] described from Slovakia have been found in the Drienovská Wetland. The detected species include potential vectors of avian haemosporidian (*C. festivipennis*, *C. alazanicus*, *C. kibunensis*, *C. segnis*, *C. punctatus*, *C. impunctatus*, *C. pictipennis*, *C. circumscriptus*, and *C. scoticus*). In this study, successful sequencing of 65 individuals of *Culicoides* demonstrated that barcoding is a useful tool for species identification. Some of the collected could not be molecularly identified to species level due to a lack of information in GenBank database. This may indicate that these species may yet to be described [51,52]. The most abundant species that was detected in the monitored area was *C. clastrieri* (31%). The DNA barcoding method, based on the *cox 1* sequence, is unable to distinguish *C. clastrieri* from *C. festivipennis* [37,53]. Those species only differ in the presence/absence of sensilla ceoloconica on their antennas. However, additional distinguishing features can be observed in the wing pattern, particularly in the number, position, and fusion of pale spots in cells m2, m, M1, and the anal cell, which allow for reliable morphological separation between the two species. They may also be differentiated by applying sPLS-DA statistical analysis, which facilitates the detection of individual cryptical species [39]. Therefore, these two species were confirmed in the present study exclusively on the basis of their morphological features, since the individual parts of the *cox 1* gene were 100% identical. The *C. obsoletus* and *C. scoticus* species are members of the Obsoletus group and are closely related species, which are difficult, or even impossible, to distinguish morphologically [54,55]. Those species were therefore diagnosed merely on the basis of the PCR assay, and all the examined biting midges in this group were found to be *C. obsoletus*. Only males of *C. obsoletus* and *C. scoticus* can be morphologically distinguished, particularly based on differences in their genital structures and other fine morphological characteristics [54].

The type of the biotope in question may significantly affect the reproduction of *Culicoides* in that area, and that eventually determines their incidence and species composition. The incidence of biting midges in different biotopes may vary [56]. The highest number of biting midges was collected on 11 and 12 June. According to Sarvašová et al. [57], mid-June is the most frequent peak of biting midge activity, during which as much as 65% of biting midges may be caught over a single night of the whole season. Follow-up studies should be conducted in other biotopes, especially in areas unaffected by humans, since there is generally a lack of information on *Culicoides* species that feed on wild animals. At present, the majority of studies on *Culicoides* species composition examine primarily farms or nearby regions where cattle are bred. These studies have often focused on the role of *Culicoides* in transmitting important livestock viruses, underscoring their significance in veterinary and epidemiological research [58].

Engorged females of *Culicoides* provide valuable information on their host preferences based on the host blood meal present in the abdomens of those vectors [59]. Since the females with fresh blood meal in their abdomens represent a relatively small portion of the total number of collected individuals, it is very difficult to collect a sufficient number of engorged females, even when *Culicoides* specimens are collected near a host or directly from the skin of potential hosts. The proportion of engorged females is always relatively low [14,60] because the females spend as much as 90% of their time digesting protein meal that is necessary for the completion of the ovarian cycle [61]. Of the 2344 collected biting midges, 46 were females with fresh blood meal in their abdomens, representing 1.96%.

The analysis of host blood from *Culicoides* revealed evident affinity to avian hosts, which is in accordance with the observations made in the surrounding European countries [14,18]. However, the use of host DNA markers for their identification has its limitations, caused by digestion of host blood and fast degradation of DNA in the midgut of insects. With an advancing stage of blood digestion, the probability of successful identification of the source of host blood may decrease [58]. Another potential reason may be the fact that the abdomens of biting midges were more or less pigmented, depending on a species, with pigmentation indicating ongoing digestion processes that can lead to the degradation of host DNA. In forty-six specimens examined for host blood, five avian species (*Turdus philomelos*, *Turdus merula*, *Coccothraustes coccothraustes*, *Pica pica*, and *Cyanistes caeruleus ogliastrae*) and three mammalian hosts (*Capreolus capreolus*, *Homo sapiens*, and *Alces alces*) were detected (Table 2). Birds were mostly parasitised by *C. clastrieri*, *C. alazanicus*, *C. festivipennis*, and *C. pictipennis.* Roe deer were parasitised by *C. obsoletus*, *C. alazanicus*, *C. clastrieri* species, and one *C. pictipennis* individual. They are regarded as opportunistic when selecting their hosts. *C. obsoletus* is especially regarded as opportunistic. However, in the present study, it was found that they prefer mammal hosts, as has been reported from other studies [12,19,20,62,63]. It should be noted that members of the Obsoletus group also feed on birds [19,60,63,64] and are one of the potential vectors of avian haemosporidian [28].

*Culicoides obsoletus* and *C. alazanicus* were confirmed to contain blood from Eurasian elk (*Alces alces*), an animal that is rare in Slovakia. Generally, only young rams are observed sporadically, as they migrate long distances looking for female partners and new regions to occupy. Typically, Eurasian elks migrate to Slovakia from Poland, where large populations can be found. In 2022, an elk was also observed in the Liptov region in central Slovakia [65]. Elks are sensitive to high temperatures in the environment and actively search for lakes and ponds [66]. They prefer enclosed forests, which mitigate their thermal stress [67]. Elks are highly mobile and their territories may extend to 10–60 km^2^ [68]. These factors may have affected the increased occurrence of elks in Slovakia, including the presence of their blood in the females of biting midges in the wetlands around Drienovec. The presence of elk blood in *Culicoides* confirms the presence of these animals in the area. It also highlights the risk of midges transmitting pathogens from the migrating animals to local populations. *Culicoides* species that feed regarding migratory birds and local populations of mammals or birds increase the risk of disease outbreaks

Host availability significantly affects how biting midges choose their hosts. Many studies have indicated that a majority of the *Culicoides* species are opportunistic and easily adapt to the hosts that are present and available in their environment, for example, the *C. circumsriptus*, *C. festivipennis*, *C. pictipennis*, and *C. punctatus* species [13,64,69,70]. However, some of the species are strictly ornithophilic (*C. duddingstoni* and *C. salinarius*) or mammophilic (*C. deltus*) [70].

The present study provides important information and knowledge of the host preferences of the examined *Culicoides* species, even though the number of specimens examined is too low to support any conclusions regarding their fundamental opportunistic or selective ornithophilic selection of hosts. Moreover, results of this study may have been affected by the fact that the light traps were installed at relatively low heights (a maximum of 2 m). That also indicates a high rate of detection of hosts of the Turdidae family, which predominantly inhabit, feed, and nest in lower strata close to the ground. Studies that examined biting midges parasitising on birds have found that it was more beneficial to install light traps at heights over 4 metres [61,64,71]. Unfortunately, in the present study, it was impossible to install the traps so high.

We used two protocols to detect avian haemosporidian parasites. The most commonly used protocol for detecting haemosporidians is based on the variability regarding the cytochrome b gene of mitochondrial DNA (*cyt b*), developed by Hellgren et al. (2004) [45]. Although, several other protocols have been created targeting different regions of *cyt b* or other genes, and various studies have compared their sensitivity and efficacy [27,45,72]. The protocol of Hellgren et al. [45] remains the gold standard for detecting avian haemosporidian, as it is the most widely used, allowing for comparability of results across studies. Due to the varying sensitivities of different PCR detection methods, as demonstrated in several studies mentioned above, we combine two distinct protocols in order to enhance the probability of detecting haemosporidians in the samples.

Haemoproteosis has previously been reported in wild birds in Slovakia, including resident species [73,74], although potential vectors were unknown. In this study, DNA of avian haemosporidian parasites was detected from *Culicoides* biting midges in Slovakia for the first time, suggesting that these insects transmit *Haemoproteus* within wetland ecosystems at the Bird Ringing Station in Drienovec.

Haemosporidian DNA was confirmed in three females with fresh blood meal. In two species, *C. alazanicus* and *C. festivipennis*, two avian blood parasites were detected *Haemoproteus tartakovskyi* (HAWF1) and *Haemoproteus asymmetricus* (TUPHI01). In one female of *C. alazanicus*, DNA of *Plasmodium matutinum* (LINN1) was confirmed (Table 3). The prevalence of pathogens in field-collected *Culicoides* is usually low and varies among species [30,31].

In Europe, Haemoproteus asymmetricus is most frequently found in Turdus merula and Turdus philomelos [9,32,75]. DNA of Haemoproteus asymmetricus of the TUPHI01 lineage was detected in biting midges of C. kibunensis, C. reconditus, C. festivipennis, and C. segnis in Lithuania [29,32,75,76]. Haemoproteus asymmetricus was described by Valkiūnas et al. [77], with the majority of reports associating it with the song thrush (Turdus philomelos). It is closely related to *Haemoproteus minutus* (TURDUS2), which is commonly found in the blackbird (*Turdus merula*) [78]. Haemoproteus tartakovskyi (HAWF1) was known to be transmitted by C. impunctatus [79] and C. segnis [30,75], and in the laboratory, this parasite in known to form sporozoites in C. nubeculosus [80].

This species of *Plasmodium* was also detected in *C. festivipennis*, *C. impunctatus*, *C. punctatus*, and *C. obsoletus* biting midges previously [29]. However, the natural vectors of this parasite are mosquitoes of the Culicidae family; therefore, abortive development of the parasites in a non-competent vector is assumed [76,81]. Non-specific avian haemosporidian are often detected in insects that feed on infected birds. For example, *Haemoproteus* was also detected in mosquitoes, while *Leucocytozoon* was detected in biting midges [82]. Eurasian thrushes and songbirds (Turdida and Passeriformes) constitute a group of birds that is most frequently infected by the *Plasmodium* species, primarily the *P. matutinum* species in the case of song thrushes (*T. philomelos*), as confirmed by studies in other European countries [78,83]. This indicates that these birds are natural reservoirs of *Plasmodium* [76].

This study suggests a potential vector for the detected avian haemosporidian parasites and provides insights into which haemosporidian species may be actively transmitted at the study site, similarly to findings reported by Guillén-Rodríguez et al. [84]. However, to confirm vector competence, it would be necessary to confirm the presence of haemosporidian stages within the salivary glands of individual *Culicoides* [22,85]. The detection of haemosporidian stages in females without any fresh blood in the abdomen will also increase the possibility that the species may act as a vector. While the detection of the parasites in recent blood meal indicates that the females fed on an infected host, the detection of the parasites in females free from visible blood may be inductive of a systemic infection. The molecular detection of haemosporidian parasites along with ornithophilic feeding behaviour of biting midges increase the potential vector capacity of these *Culicoides* species. This study contributes new data that enrich our current understanding of the relationships between avian haemosporidians and their potential vectors of the *Culicoides* genus. Nonetheless, to definitively determine which haemosporidian lineages are actively transmitted in Slovakia, further research is required. The promising results of this study motivate continued investigation, with the aim of broadening the analysis of both vector and avian host communities at the site. This integrative approach will help provide a more comprehensive understanding of parasite transmission dynamics, which remains a primary goal for our future research.

## 5. Conclusions

The present study reports the occurrence of avian and mammalian blood parasites in the vectors of *Culicoides* spp. in the Bird Ringing Station in Drienovec, eastern Slovakia. The analysis of the origin of host blood obtained from *Culicoides* provides valuable information in epidemiological and ecological studies. The current knowledge, based on molecular studies, indicates that the host preferences significantly vary among the *Culicoides* females. This leads to the potential amplification and transmission of pathogens between a vector and a host. This is the first detection of avian haemosporidian DNA in *Culicoides* vectors in Slovakia. Specifically, it was identified in females containing fresh host blood in their abdomens, indicating potential transmission across the monitored area.

Since the present study did not confirm the sporogonic transmission of parasites, further research is necessary to identify the competent vectors of these blood parasites. The present study contributes to the epizootiologic knowledge of the spread of avian infections caused by *Haemoproteus* by specifying the *Culicoides* species as the vectors and species that are probably responsible for their transmission in Europe.

MalAvi database remains a reliable and indispensable resource for molecular genetic studies of avian haemosporidians, as it compiles comprehensive data on all identified parasite lineages along with the relevant literature. Nonetheless, the authors also address persisting limitations regarding the availability of sequence data for comparative analyses and underscore the necessity of depositing all such data in GenBank to ensure transparency and reproducibility.

## Figures and Tables

**Table 1:** Molecular identification of *Culicoides* species using DNA barcoding. Specimens were collected at two sites (i.e., a pond and a floodplain forest) using CDC miniature UV light traps, operated from 4 June to 30 July 2022 at the Bird Ringing Station in Drienovec.

*Sample Number*	*Site*	*Collection Date*	*Molecular Identification*	*Physiological State*	*GenBank*	*Highest Sequence Identity with GenBank Database*		
						Identity	Accession Number	
*N44Dj*	Pond	29.-30.7.	*C. obsoletus*	Blood-fed	PV055075	**100%**	MW633530.1	
*N46Dj*	Pond	29.-30.7.	*C. alazanicus*	Blood-fed	PV055076	**100%**	KJ624068.1	
*N47Dj*	Pond	29.-30.7.	*C. alazanicus*	Blood-fed	PV055077	**99.80%**	KJ624068.1	
*N48Dj*	Pond	29.-30.7.	*C. deltus*	Unfed	PV055078	**100%**	KJ624075.1	
*N63Dl*	Floodplain forest	29.-30.7.	*C. alazanicus*	Blood-fed	PV055079	**100%**	KJ624068.1	
*N64Dl*	Floodplain forest	29.-30.7.	*C. alazanicus*	Blood-fed	PV055080	**100%**	KJ624068.1	
*N65Dj*	Pond	22.-23.6.	*C. obsoletus*	Blood-fed	PV055081	**100%**	OQ941538.1	
*N66Dj*	Pond	22.-23.6.	*C. obsoletus*	Blood-fed	PV055082	**99.79%**	JQ620141.1	
*N67Dj*	Pond	22.-23.6.	*C. clastrieri*	Blood-fed	PV055083	**100%**	MW353349.1	
*N68Dj*	Pond	22.-23.6.	*C. segnis*	Gravid	PV055084	**99.78%**	KY707778.1	
*N69Dj*	Pond	22.-23.6.	*Culicoides* sp.	Gravid	PV055085	**87.47%**	KY433537.1	*C. arakawae*
*N70Dj*	Pond	22.-23.6.	*C. kibunensis*	Gravid	PV055086	**100%**	KJ624096.1	
*N71Dj*	Pond	22.-23.6.	*Culicoides* sp.	Gravid	PV055087	**87.47%**	KY433537.1	*C. arakawae*
*N72Dj*	Pond	22.-23.6.	*C. segnis*	Gravid	PV055088	**99.78%**	KY707778.1	
*N73Dj*	Pond	22.-23.6.	*C. segnis*	Gravid	PV055089	**100%**	KJ624127.1	
*N74Dj*	Pond	7.-8.7.	*C. pallidicornis*	Gravid	PV055090	**99.58%**	JQ620149.1	
*N75Dj*	Pond	7.-8.7.	*C. segnis*	Gravid	PV055091	**100%**	KJ624127.1	
*N76Dj*	Pond	7.-8.7.	*C. segnis*	Gravid	PV055092	**100%**	KJ624127.1	
*N77Dj*	Pond	7.-8.7.	*C. segnis*	Gravid	PV055093	**100%**	KJ624127.1	
*N78Dj*	Pond	7.-8.7.	*C. kibunensis*	Gravid	PV055094	**100%**	KJ624096.1	
*N79Dj*	Pond	7.-8.7.	*C. kibunensis*	Gravid	PV055095	**99.53%**	JQ620101.1	
*N80Dj*	Pond	7.-8.7.	*Culicoides* sp.	Gravid	PV055096	**87.47%**	KY433537.1	*C. arakawae*
*N81Dj*	Pond	7.-8.7.	*C. shaklawensis*	Gravid	PV055097	**99.80%**	KJ624129.1	
*N82Dl*	Floodplain forest	29.-30.7.	*C. clastrieri*	Gravid	PV055098	**100%**	MW353349.1	
*N83Dl*	Floodplain forest	29.-30.7.	*C. clastrieri*	Gravid	PV055099	**100%**	MW353349.1	
*N124Dl*	Floodplain forest	11.-12.6.	*C. kibunensis*	Gravid	PV055100	**100%**	KJ624096.1	
*N125Dl*	Floodplain forest	11.-12.6.	*Culicoides* sp.	Parous	PV055101	**87.47%**	KY433537.1	*C. arakawae*
*N126Dl*	Floodplain forest	11.-12.6.	*C. kibunensis*	Parous	PV055102	**100%**	KJ624096.1	
*N133Dl*	Floodplain forest	11.-12.6.	*C. pallidicornis*	Parous	PV055103	**100%**	JQ620149.1	
*N136Dl*	Floodplain forest	11.-12.6.	*C. obsoletus*	Blood-fed	PV055104	**100%**	MW633838.1	
*N137Dl*	Floodplain forest	11.-12.6.	*Culicoides* sp.	Parous	PV055105	**87.47%**	KY433537.1	*C. arakawae*
*N138Dl*	Floodplain forest	11.-12.6.	*C. pictipennis*	Blood-fed	PV055106	**100%**	MW353287.1	
*N139Dl*	Floodplain forest	11.-12.6.	*C. obsoletus*	Blood-fed	PV055107	**100%**	MW633838.1	
*N140Dl*	Floodplain forest	11.-12.6.	*C. alazanicus*	Blood-fed	PV055108	**100%**	KJ624068.1	
*N141Dl*	Floodplain forest	11.-12.6.	*C. obsoletus*	Blood-fed	PV055109	**100%**	OM665428.1	
*N142Dl*	Floodplain forest	11.-12.6.	*C. obsoletus*	Blood-fed	PV055110	**100%**	OM665428.1	
*N143Dl*	Floodplain forest	11.-12.6.	*C. obsoletus*	Blood-fed	PV055111	**100%**	OM665428.1	
*N144Dl*	Floodplain forest	11.-12.6.	*C. obsoletus*	Blood-fed	PV055112	**100%**	MW633838.1	
*N145Dl*	Floodplain forest	11.-12.6.	*C. clastrieri*	Blood-fed	PV055113	**100%**	MW353349.1	
*N146Dl*	Floodplain forest	11.-12.6.	*C. clastrieri*	Blood-fed	PV055114	**100%**	MW353349.1	
*N147Dl*	Floodplain forest	11.-12.6.	*C. clastrieri*	Blood-fed	PV055115	**100%**	MW353349.1	
*N148Dl*	Floodplain forest	11.-12.6.	*C. clastrieri*	Blood-fed	PV055116	**100%**	MW353349.1	
*N149Dl*	Floodplain forest	11.-12.6.	*C. clastrieri*	Blood-fed	PV055117	**100%**	MW353349.1	
*N150Dl*	Floodplain forest	11.-12.6.	*C. pallidicornis*	Parous	PV055118	**100%**	JQ620149.1	
*N152Dj*	Pond	11.-12.6.	*C. festivipennis*	Blood-fed	PV055119	**100%**	OM665438.1	
*N153Dj*	Pond	11.-12.6.	*C. alazanicus*	Blood-fed	PV055120	**100%**	KJ624068.1	
*N154Dj*	Pond	11.-12.6.	*C. festivipennis*	Blood-fed	PV055121	**99.60%**	OM665449.1	
*N155Dj*	Pond	11.-12.6.	*C. alazanicus*	Blood-fed	PV055122	**99.80%**	KJ624068.1	
*N156Dj*	Pond	11.-12.6.	*C. clastrieri*	Blood-fed	PV055123	**100%**	MW353349.1	
*N157Dj*	Pond	11.-12.6.	*C. alazanicus*	Blood-fed	PV055124	**99.80%**	KJ624068.1	
*N158Dj*	Pond	11.-12.6.	*C. alazanicus*	Blood-fed	PV055125	**100%**	KJ624068.1	
*N159Dj*	Pond	11.-12.6.	*C. alazanicus*	Blood-fed	PV055126	**100%**	KJ624068.1	
*N163Dj*	Pond	11.-12.6.	*C. newsteadi*	Blood-fed	PV055127	**99.80%**	MW642500.1	
*N164Dl*	Floodplain forest	11.-12.6.	*C. clastrieri*	Blood-fed	PV055128	**100%**	MW353349.1	
*N165Dl*	Floodplain forest	11.-12.6.	*Culicoides* sp.	Blood-fed	PV055129	**86.96%**	JN545047.1	*C.oxystoma*
*N167Dj*	Pond	11.-12.6.	*C. obsoletus*	Blood-fed	PV055130	**99.80%**	MW642205.1	
*N168Dl*	Floodplain forest	11.-12.6.	*C. clastrieri*	Blood-fed	PV055131	**100%**	MW353349.1	
*N169Dj*	Pond	11.-12.6.	*C. clastrieri*	Blood-fed	PV055132	**100%**	MW353349.1	
*N171Dl*	Floodplain forest	29.-30.7.	*C. obsoletus*	Blood-fed	PV055133	**100%**	MW633284.1	
*N176Dl*	Floodplain forest	4.-5.6.	*C. segnis*	Parous	PV055134	**100%**	KJ624128.1	
*N177Dl*	Floodplain forest	4.-5.6.	*C. segnis*	Parous	PV055135	**100%**	KJ624128.1	
*N181Dj*	Pond	11.-12.6	*C. pictipennis*	Parous	PV055136	**99.76%**	KJ624113.1	
*N183Dj*	Pond	11.-12.6	*C. segnis*	Parous	PV055137	**99.60%**	KJ624128.1	
*N256Dl*	Floodplain forest	22.-23.6.	*C. festivipennis*	Blood-fed	PV055138	**100%**	OM665438.1	
*N257Dl*	Floodplain forest	22.-23.6.	*C. clastrieri*	Blood-fed	PV055139	**100%**	MW353349.1	

**Table 2:** Host preferences based on blood meals from engorged females of *Culicoides* species **(*n* = 36)** collected at the Bird Ringing Station in Drienovec, with “***n***” indicating the number of individuals detected per host species.

	*C. obsoletus* (*n* = 9)	*C. clastrieri* (*n* = 10)	*C. alazanicus* (*n* = 10)	*C. festivipennis* (*n* = 3)	*C. pictipennis* (*n* = 2)	*Culicoides* spp. (*n* = 2)
**Mammals**						
*Capreolus capreolus*	8	4	1	1	1	-
*Homo sapiens*	-	3	1	-	-	2
*Alces alces*	1	-	1	-	-	-
**Birds**						
Turdidae (family)	-	-	2	-	-	-
*Turdus philomelos*	-	1	3	-		-
*Turdus merula*	-	2	1	-	1	-
*Pica pica*	-	-		1	-	-
*Cyanistes caeruleus ogliastrae*	-	-	1	-	-	-
*Coccothraustes coccothraustes*	-	-	-	1	-	-

**Table 3:** Summary of blood-fed *Culicoides* females, including molecular species identification, host species, and associated haemosporidian parasites with their genetic lineages.

Sample Number	*Culicoides* Species	Site	Host Blood	Primers	Parasite Species	Genetic Lineage
N46Dj	*C. alazanicus*	Pond	Turdidae family	HeamF/HeamR2	*Plasmodium matutinum*	LINN1
N64Dl	*C. alazanicus*	Floodplain forest	*Turdus philomelos*	HeamF/HeamR2	*Haemoproteus asymmetricus*	TUPHI01
N256Dl	*C. festivipennis*	Floodplain forest	*Coccothraustes* *coccothraustes*	DW1/DW6	*Haemoproteus tartakovskyi*	HAWF1

## Data Availability

All data presented in this article are available in the manuscript in tables and figures. The respective data can be found in GenBank at: https://www.ncbi.nlm.nih.gov/genbank/ under the numbers PV055075-PV055139.
